# Explainable machine learning for predicting distant metastases in renal cell carcinoma patients: a population-based retrospective study

**DOI:** 10.3389/fmed.2025.1624198

**Published:** 2025-07-29

**Authors:** Zhao Hou, Peipei Wang, Dingyang Lv, Huiyu Zhou, Zhiwei Guo, Jinshuai Li, Mohan Jia, Hongyang Du, Weibing Shuang

**Affiliations:** ^1^Department of Urology, The First Hospital of Shanxi Medical University, Taiyuan, Shanxi, China; ^2^Academy of Medical Sciences, Shanxi Medical University, Taiyuan, Shanxi, China; ^3^School of Public Health, Shanxi Medical University, Taiyuan, Shanxi, China; ^4^Department of First Clinical Medical College, Shanxi Medical University, Taiyuan, Shanxi, China

**Keywords:** renal cell carcinoma, distant metastasis, machine learning, predictive modeling, external validation, web-based calculator

## Abstract

**Background:**

Distant metastasis is a key factor contributing to poor prognosis in renal cell carcinoma (RCC). Early prediction of metastasis is crucial for developing personalized treatment plans and improving patient outcomes. This study aimed to establish and validate a clinical prediction model for distant metastasis in RCC patients.

**Methods:**

Ten machine learning algorithms were employed to develop a predictive model for distant metastasis in RCC. Data from 51,566 RCC patients in The Surveillance, Epidemiology, and End Results (SEER) database (2010–2018) were used for model development, while 726 RCC patients from the First Hospital of Shanxi Medical University were selected for external validation. Hyperparameters were optimized using grid search and tenfold cross-validation. Model performance was assessed using metrics such as the area under the receiver operating characteristic curve (AUC), the area under the precision-recall curve (AUPRC), decision curve analysis, calibration curves, precision, and accuracy. Shapley additive explanations (SHAP) were used for model interpretation. The best-performing model was then used to create a web-based calculator to predict metastasis risk in RCC patients.

**Results:**

The study included 51,566 RCC patients, with 3,667 showing distant metastases. Logistic regression identified tumor size, grade, T-stage, N-stage, radiotherapy, chemotherapy, and surgery as independent risk factors. The Extreme Gradient Boosting (XGB) model demonstrated superior performance (AUC: 0.957, Accuracy: 0.898) in the training set and was validated externally (AUC: 0.742, Accuracy: 0.904). A web-based calculator was developed using the XGB model.

**Conclusion:**

This study designed and validated an XGB model using clinicopathologic data to predict the risk of distant metastasis in RCC patients, potentially aiding clinical decision-making.

## 1 Introduction

Renal cell carcinoma is the 14th most common malignancy worldwide, with over 430,000 new cases reported in 2020, and is the most common histopathological subtype, constituting approximately 90% of all renal malignancies (1). According to relevant epidemiological evidence, renal cancer is the ninth most diagnosed cancer in female patients and the sixth most diagnosed in male patients, accounting for 3% and 5% of all malignant tumor diagnoses, respectively (2). Despite the increase in its incidence, overall mortality from RCC has been decreasing (3, 4). Advances in therapeutic strategies such as targeted therapies and immune checkpoint inhibitors (ICIs) have led to an improvement in the prognosis of patients with RCC (5), but there is a significant difference in the prognosis of patients with limited and metastatic renal cancer. The 5 years survival rate is nearly 93% for limited renal cancer and only 17% for patients with distant metastases (2, 6). Previous studies have shown that 18%–30% of RCC patients present with systemic metastases at initial diagnosis, and an additional one-third develop metastatic disease following nephrectomy during long-term follow-up (7, 8). Among metastatic RCC (mRCC) cases, approximately 75% exhibit three or more metastatic locations (9). Therefore, identifying risk factors for RCC metastasis, as well as developing metastasis prediction models, is essential for improving the survival prospects of patients with RCC.

Artificial Intelligence (AI) is a branch of computer science focused on creating systems capable of performing tasks that typically require human intelligence (10). Machine learning (ML) is at the heart of AI, which uses algorithms to enable machines to train or learn from large amounts of empirical data without specific computer programming, to generate patterns to form corresponding models, and iteratively refine predictive models without explicit programming (11). Traditional statistical methods emphasize hypothesis testing and causal inference under rigid parametric assumptions, which inherently constrains their capacity to enhance predictive accuracy and generalizability in complex, real-world clinical scenarios. Furthermore, their reliance on manual feature engineering and linearity assumptions fundamentally limits scalability when analyzing high-dimensional biomedical data or unstructured clinical data streams (12). ML can integrate computer science and statistics with medical problems, and its use of complex algorithms running on large-scale, heterogeneous datasets can be used to discover useful models. As summarized by Suarez-Ibarrola et al. (13) ML and Deep Learning (DL) were found to outperform traditional statistical methods in diagnosis, prediction of response to treatment, prediction of pathology grading, and patient survival in urological disorders such as urolithiasis, renal cancer, prostate cancer, and bladder cancer.

In this study, ten ML predictive models were developed based on conventional clinicopathological parameters to identify the key factors influencing distant metastasis in RCC. The performance of these models was comprehensively evaluated using multiple metrics, and the interpretability of their key features was thoroughly addressed. Ultimately, the optimal models were integrated into a clinical practice framework to assist in the screening of high-risk patients, thereby improving the accuracy of the diagnosis of distant metastasis of RCC and providing an evidence-based basis for the development of therapeutic guidelines and standards of care.

## 2 Materials and methods

### 2.1 Data source and patient cohorts

**FIGURE 1:**
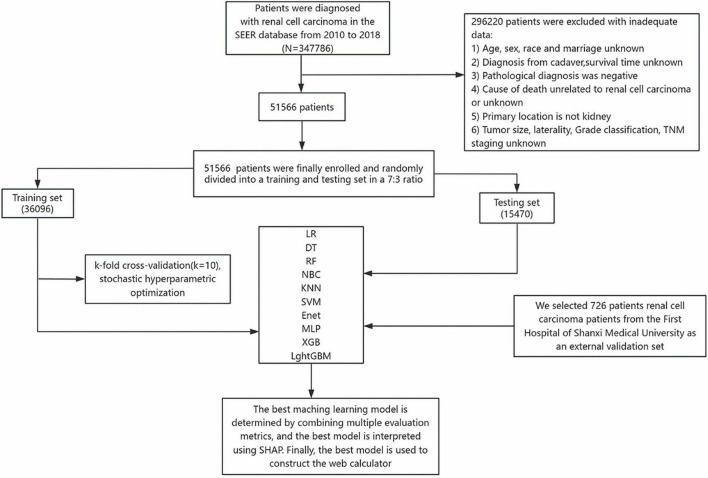
Study design and patient screening workflow diagram.

This study used a retrospective cohort design with data from the Surveillance, Epidemiology, and End Results (SEER) database (2010–2018) established by the National Cancer Institute and an independent validation cohort at the First Hospital of Shanxi Medical University (2013–2021) The SEER database covers 28% of the United States population and provides us with a large amount of data from cancer-related research, and information on metastatic tumors has been systematically collected since 2010 (14). Patients with RCC meeting the following criteria were extracted by SEER*STAT 8.4.4 software. Inclusion criteria: histologically confirmed primary RCC (International Classification of Diseases of Oncology ICD-O-3 codes: 8120/3 for migratory cell carcinoma, 8130/3 for papillary migratory cell carcinoma, 8260/3 for papillary adenocarcinoma, 8310/3 for clear cell adenocarcinoma, 8312/3 for renal cell carcinoma, 8317/3 for chromophobe cell carcinoma) Exclusion criteria: (1) missing demographic/tumor characteristics (age, sex, tumor size, TNM stage, etc.); (2) autopsy-confirmed diagnosis; and (3) unknown survival time or cause of death. A total of 51,566 patients from the SEER cohort were ultimately included. The data were divided into training and testing sets in a 7:3 ratio, with 726 patients from a single-center cohort in China used for external validation. The study variables included three major dimensions: demographic characteristics (age, gender, race, and marital status), tumor characteristics: (size, histological subtype, laterality, grading, and T/N/M staging), and treatment modalities: (surgery, radiotherapy, and chemotherapy). This study followed the Declaration of Helsinki, SEER data were granted an ethical exemption due to de-identified characteristics (open access number)^1^, and the external validation cohort received approval from the Ethics Committee of the First Hospital of Shanxi Medical University (approval number: 2018 K006). Specific information about the SEER and external validation of the RCC cohort is shown in Table 1. The study flow of this paper is shown in Figure 1.

**TABLE 1:** Characterization of clinical and pathological data in the training, test, and validation cohort.

Variables	SEER database (*N* = 51,566)		External validation (*N* = 726)	*P*-value
	Train (*N* = 36,096)	Test (*N* = 15,470)		
**Age, n (%)**				
< 50	6,882 (19.1)	3,001 (19.4)	48 (6.6)	*P* < 0.001
50–60	9,698 (26.9)	4,004 (25.9)	78 (10.7)	–
60–70	11,378 (31.5)	4,984 (32.2)	67 (9.2)	–
≥ 70	8,138 (22.5)	3,481 (22.5)	533 (73.4)	–
**Sex, n (%)**				
Male	22,802 (63.2)	9,833 (63.6)	468 (64.5)	*P* = 0.566
Female	13,294 (36.8)	5,637 (36.4)	258 (35.5)	–
**Race, n (%)**				
White	29,854 (82.7)	12,876 (83.2)	0 (0.0)	*P* < 0.001
Black	3,656 (10.1)	1,544 (10.0)	0 (0.0)	–
American Indian/Alaska Native	368 (1.0)	146 (0.9)	0 (0.0)	–
Asian or Pacific Islander	2,218 (6.1)	904 (5.8)	726 (100.0)	–
**Marital, n (%)**				
Single (never married)	5,850 (16.2)	2,508 (16.2)	5 (0.7)	*P* < 0.001
Married (including common law)	23,719 (65.7)	10,255 (66.3)	707 (97.4)	–
Separated	440 (1.2)	173 (1.1)	0 (0.0)	–
Divorced	3,452 (9.6)	1,462 (9.5)	5 (0.7)	–
Widowed	2,487 (6.9)	1,012 (6.5)	9 (1.2)	–
Unmarried or domestic partner	148 (0.4)	60 (0.4)	0 (0.0)	–
**Size, n (%)**				
≤ 5	22,307 (61.8)	9,602 (62.1)	174 (24.0)	*P* < 0.001
> 5	13,789 (38.2)	5,868 (37.9)	552 (76.0)	–
**Laterality, n (%)**				
Right	18,330 (50.8)	7,889 (51.0)	382 (52.6)	*P* = 0.578
Left	17,766 (49.2)	7,581 (49.0)	344 (47.4)	–
**Tumor histology, n (%)**				
8,120/3	162 (0.4)	63 (0.4)	0 (0.0)	*P* < 0.001
8,130/3	107 (0.3)	43 (0.3)	0 (0.0)	–
8,260/3	4,693 (13.0)	1,949 (12.6)	17 (2.3)	–
8,310/3	25,466 (70.6)	10,990 (71.0)	678 (93.4)	–
8,312/3	4,067 (11.3)	1,758 (11.4)	13 (1.8)	–
8,317/3	1,601 (4.4)	667 (4.3)	18 (2.5)	–
**Grade, n (%)**				
Grade I	4,094 (11.3)	1,750 (11.3)	125 (17.2)	*P* < 0.001
Grade II	18,728 (51.9)	8,179 (52.9)	430 (59.2)	–
Grade III	10,655 (29.5)	4,421 (28.6)	149 (20.5)	–
Grade IV	2,619 (7.3)	1,120 (7.2)	22 (3.0)	–
**T-stage, n (%)**				
T1	24,701 (68.4)	10,551 (68.2)	632 (87.1)	*P* < 0.001
T2	3,700 (10.3)	1,593 (10.3)	70 (9.6)	–
T3	7,290 (20.2)	3,142 (20.3)	20 (2.8)	–
T4	405 (1.1)	184 (1.2)	4 (0.6)	–
**N-stage, n (%)**				
N0	34,800 (96.4)	14,897 (96.3)	710 (97.8)	*P* = 0.047
N1	907 (2.5)	414 (2.7)	16 (2.2)	–
N2	389 (1.1)	159 (1.0)	0 (0.0)	–
**M-stage, n (%)**				
M0	33,537 (92.9)	14,362 (92.8)	644 (88.7)	*P* < 0.001
M1	2,559 (7.1)	1,108 (7.2)	82 (11.3)	–
**Radiation, n (%)**				
No	35,298 (97.8)	15,121 (97.7)	716 (98.6)	*P* = 0.29
Yes	798 (2.2)	349 (2.3)	10 (1.4)	–
**Chemotherapy, n (%)**				
No	34,075 (94.4)	146,16 (94.5)	700 (96.4)	*P* = 0.062
Yes	2,021 (5.6)	854 (5.5)	26 (3.6)	–
**RX Summ-Surg, n (%)**				
No	1,792 (5.0)	740 (4.8)	0 (0.0)	*P* < 0.001
Yes	34,304 (95.0)	14,730 (95.2)	726 (100.0)	–

RX Summ-Surg, surgery.

### 2.2 Feature screening

This study employed LASSO regression for feature dimensionality reduction to select candidate variables. Subsequently, univariate logistic regression was performed to preliminarily identify potential predictors associated with metastasis. Multivariate logistic regression was then used to determine the independent risk factors for distant metastasis in RCC (*P* < 0.05). These key variables were subsequently incorporated into the machine-learning modeling process.

### 2.3 Model development and evaluation

This study employs ten machine learning algorithms: Logistic Regression (LR) (15), Decision Trees (DT) (16), Random Forests (RF) (17), Naive Bayes (NBC) (18), K Nearest Neighbors (KNN) (19), Support Vector Machines (SVM) (20), Elastic Networks (Enet) (21), Multilayer Perceptrons (MLP) (22), Extreme Gradient Boosting (XGB) (23), Lightweight Gradient Boosting Machine (LightGBM) (24).

The models were developed using a training dataset. Notably, Logistic Regression was evaluated using 10-fold cross-validation but did not require hyperparameter tuning due to its straightforward nature. Conversely, grid search hyperparameter tuning was conducted for the remaining nine machine learning algorithms, building upon the results from the 10-fold cross-validation, to ensure optimal performance and mitigate the risk of overfitting. The specifics of the hyperparameter optimizations are as follows:

Decision Tree (DT): Parameters optimized included the Cost-Complexity Parameter (cost_complexity), maximum depth of the tree (tree_depth), and the minimum number of data points in a node (min_n).

Random Forest (RF): Optimization focused on the number of features randomly selected for splitting (mtry), the number of trees (trees), and the minimum number of data points in a node (min_n).

Naive Bayes (NBC): The relative smoothness of the class boundary (smoothness) and the Laplace correction parameter (Laplace) were optimized.

K Nearest Neighbors (KNN): A single integer representing the number of neighbors to consider (neighbors) was optimized.

Support Vector Machines (SVM): Parameters optimized included the cost of predicting samples within or beyond the margin (cost) and the sigma value for the Radial Basis Function (rbf_sigma).

Elastic Networks (Enet): Optimized parameters included the amount of regularization (penalty) and the proportion of Lasso Penalty (mixture).

Multilayer Perceptrons (MLP): Key parameters optimized were the number of units in the hidden layer (hidden_units), the amount of regularization (penalty), and the number of training iterations (epochs).

Extreme Gradient Boosting (XGB): The optimization process included the number of predictors randomly sampled at each split (mtry), maximum depth of the tree (tree_depth), minimum number of data points in a node (min_n), learning rate (learn_rate), loss reduction required for additional splits (loss_reduction), and size of the dataset exposed during fitting (sample_size).

Lightweight Gradient Boosting Machine (LightGBM): The number of predictors sampled at each split (mtry), the number of trees in model training (trees), minimum number of data points in a node (min_n), maximum depth of the tree (tree_depth), learning rate (learn_rate), and minimum loss reduction (loss_reduction) were all subjects of optimization. The specific hyperparameter values for each model are provided in Supplementary Table 1.

To assess the generalization ability of the models, the ten developed models were applied to both the internal test set and external validation set. The performance was comprehensively evaluated using receiver operating characteristic (ROC) curves, Precision-Recall (PR) curves, calibration curves, and confusion matrix results on the training set, internal test set, and external validation set. The model with the best performance was selected based on the relevant metrics.

Additionally, Shapley Additive Explanations (SHAP), a model-agnostic interpretability technique based on cooperative game theory, was employed to explain the predictions made by the best-performing ensemble machine learning model (25). The SHAP method was used to calculate the importance of each variable in the optimal model. Finally, we constructed a network calculator to facilitate the generalization and application of the model in clinical settings.

### 2.4 Statistical analysis

Data analysis was performed using R software (version 4.2.2). Due to the marked imbalance in the number of RCC patients with distant metastases compared to those without, we applied the Synthetic Minority Over-sampling Technique (SMOTE) to increase the number of patients with distant metastases, thereby mitigating the impact of class imbalance on model performance. SMOTE generates synthetic samples and incorporates them into the minority class to address the imbalance in the original dataset, ultimately improving model accuracy (26). Chi-square tests and Fisher’s exact tests were used to compare categorical variables between different groups, with categorical variables reported as frequency (percentage, %). A *P*-value less than 0.05 was considered statistically significant.

## 3 Result

### 3.1 Baseline characteristics of the study cohort

A total of 51,566 RCC patients were included in the study, sourced from the SEER database. Of these, 3,667 (7.11%) developed distant metastases and 47,899 (92.89%) did not. Table 2 presents the demographic and clinicopathological characteristics of all the patients included in the study. Patients from the SEER database were randomly assigned to a training set (*n* = 36,096) and an internal test set (*n* = 15,470) in a 7:3 ratio. External validation was conducted using data from 736 RCC patients at the First Hospital of Shanxi Medical University (Table 3). Detailed information on the training, testing, and validation cohorts is provided in Table 1.

**TABLE 2:** Overview of clinical and pathological characteristics of the Surveillance, Epidemiology, and End Results (SEER) database cohort.

Variables	SEER cohort			*P*-value
	All (*N* = 51566)	DM (−) (*N* = 47899)	DM (+) (*N* = 3667)	
**Age, n (%)**				
< 50	9,883 (19.17)	9,455 (19.74)	428 (11.67)	*P* < 0.0001
50–60	13,702 (26.57)	12,700 (26.51)	1,002 (27.32)	–
60–70	16,362 (31.73)	15,088 (31.50)	1,274 (34.74)	–
≥ 70	11,619 (22.53)	10,656 (22.25)	963 (26.26)	–
**Sex, n (%)**				
Male	32,635 (63.29)	30,096 (62.83)	2,539 (69.24)	*P* < 0.0001
Female	18,931 (36.71)	17,803 (37.17)	1,128 (30.76)	–
**Race, n (%)**				
White	42,730 (82.86)	39,611 (82.70)	3,119 (85.06)	*P* < 0.0001
Black	5,200 (10.08)	4,941 (10.32)	259 (7.06)	–
American Indian/Alaska Native	514 (1.00)	478 (1.00)	36 (0.98)	–
Asian or Pacific Islander	3,122 (6.05)	2,869 (5.99)	253 (6.90)	–
**Marital, n (%)**				
Single (never married)	83 58 (16.21)	7,801 (16.29)	557 (15.19)	*P* = 0.0131
Married (including common law)	33 974 (65.88)	31,581 (65.93)	2,393 (65.26)	–
Separated	613 (1.19)	579 (1.21)	34 (0.93)	–
Divorced	4 914 (9.53)	4 530 (9.46)	384 (10.47)	–
Widowed	3 499 (6.79)	3 214 (6.71)	285 (7.77)	–
Unmarried or domestic partner	208 (0.40)	194 (0.41)	14 (0.38)	–
**Size, n (%)**				
≤ 5	31 909 (61.88)	31 446 (65.65)	463 (12.63)	*P* < 0.0001
> 5	19 657 (38.12)	16 453 (34.35)	3,204 (87.37)	–
**Laterality, n (%)**				
Right	26 219 (50.85)	24 479 (51.11)	1 740 (47.45)	*P* < 0.0001
Left	25 347 (49.15)	23 420 (48.89)	1 927 (52.55)	–
**Tumor histology, n (%)**				
8,120/3	225 (0.44)	155 (0.32)	70 (1.91)	*P* < 0.0001
8,130/3	150 (0.29)	136 (0.28)	14 (0.38)	–
8,260/3	6,642 (12.88)	6,430 (13.42)	212 (5.78)	–
8,310/3	36,456 (70.70)	33,771 (70.50)	2,685 (73.22)	–
8,312/3	5,825 (11.30)	5,180 (10.81)	645 (17.59)	–
8,317/3	2,268 (4.39)	2,227 (4.65)	41 (1.12)	–
**Grade, n (%)**				
Grade I	5,844 (11.33)	5,738 (11.98)	106 (2.89)	*P* < 0.0001
Grade II	26,907 (52.18)	26,097 (54.48)	810 (22.09)	–
Grade III	15,076 (29.24)	13,492 (28.17)	1,584 (43.20)	–
Grade IV	3,739 (7.25)	2,572 (5.37)	1,167 (31.82)	–
**T-stage, n (%)**				
T1	35,252 (68.36)	34,682 (72.41)	570 (15.54)	*P* < 0.0001
T2	5,293 (10.26)	4,657 (9.72)	636 (17.34)	–
T3	10,432 (20.23)	8,338 (17.41)	2,094 (57.10)	–
T4	589 (1.14)	222 (0.46)	367 (10.01)	–
**N-stage,*n* (%)**				
N0	49,697 (96.38)	47,119 (98.37)	2,578 (70.30)	*P* < 0.0001
N1	1,321 (2.56)	587 (1.23)	734 (20.02)	–
N2	548 (1.06)	193 (0.40)	355 (9.68)	–
**Radiation, n (%)**				
No	50,419 (97.78)	47,740 (99.67)	2,679 (73.06)	*P* < 0.0001
Yes	1,147 (2.22)	159 (0.33)	988 (26.94)	–
**Chemotherapy, n (%)**				
No	48,691 (94.42)	47,058 (98.24)	1,633 (44.53)	*P* < 0.0001
Yes	2,875 (5.58)	841 (1.76)	2,034 (55.47)	–
**RX Summ-Surg, n (%)**				
No	2,532 (4.91)	1,759 (3.67)	773 (21.08)	*P* < 0.0001
Yes	49,034 (95.09)	46,140 (96.33)	2,894 (78.92)	–

DM (+), patients with distant metastasis; DM (−), patients without distant metastasis; RX Summ-Surg, surgery.

**TABLE 3:** Clinical and pathological characteristics of the Chinese cohort study population.

Variables	Chinese cohort			*P*-value
	All (*N* = 726)	DM (−) (*N* = 644)	DM (+) (*N* = 82)	
**Age, n (%)**				
< 50	48 (6.61)	43 (6.68)	5 (6.10)	*P* = 0.1107
50–60	78 (10.74)	67 (10.40)	11 (13.41)	–
60–70	67 (9.23)	54 (8.39)	13 (15.85)	–
≥ 70	533 (73.42)	480 (74.53)	53 (64.63)	–
**Sex, n (%)**				
Male	468 (64.46)	407 (63.20)	61 (74.39)	*P* = 0.0612
Female	258 (35.54)	237 (36.80)	21 (25.61)	–
**Race, n (%)**				
White	0	0	0	NA
Black	0	0	0	–
American Indian/Alaska Native	0	0	0	–
Asian or Pacific Islander	726 (100.00)	644 (100.00)	82 (100.00)	–
**Marital, n (%)**				
Single (never married)	5 (0.69)	5 (0.78)	0 (0.00)	*P* = 0.1168
Married (including common law)	707 (97.38)	627 (97.36)	80 (97.56)	–
Separated	0	0	0	–
Divorced	5 (0.69)	3 (0.47)	2 (2.44)	–
Widowed	9 (1.24)	9 (1.40)	0 (0.00)	–
Unmarried or domestic partner	0	0	0	–
**Size, n (%)**				
≤ 5	174 (23.97)	153 (23.76)	21 (25.61)	*P* = 0.816
> 5	552 (76.03)	491 (76.24)	61 (74.39)	–
**Laterality, n (%)**				
Right	382 (52.62)	341 (52.95)	41 (50.00)	*P* = 0.6991
Left	344 (47.38)	303 (47.05)	41 (50.00)	–
**Tumor histology, n (%)**				
8,120/3	0	0	0	*P* = 0.2086
8,130/3	0	0	0	–
8,260/3	17 (2.34)	16 (2.48)	1 (1.22)	–
8,310/3	678 (93.39)	604 (93.79)	74 (90.24)	–
8,312/3	13 (1.79)	10 (1.55)	3 (3.66)	–
8,317/3	18 (2.48)	14 (2.17)	4 (4.88)	–
**Grade, n (%)**				
Grade I	125 (17.22)	119 (18.48)	6 (7.32)	*P* = 0.0001
Grade II	430 (59.23)	388 (60.25)	42 (51.22)	–
Grade III	149 (20.52)	122 (18.94)	27 (32.93)	–
Grade IV	22 (3.03)	15 (2.33)	7 (8.54)	–
**T-stage, n (%)**				
T1	632 (87.05)	579 (89.91)	53 (64.63)	*P* < 0.0001
T2	70 (9.64)	50 (7.76)	20 (24.39)	–
T3	20 (2.76)	14 (2.17)	6 (7.32)	–
T4	4 (0.55)	1 (0.16)	3 (3.66)	–
**N-stage, n (%)**				
N0	710 (97.80)	642 (99.69)	68 (82.93)	*P* < 0.0001
N1	16 (2.20)	2 (0.31)	14 (17.07)	–
N2	0	0	0	–
**Radiation, n (%)**				
No	716 (98.62)	643 (99.84)	73 (89.02)	*P* < 0.0001
Yes	10 (1.38)	1 (0.16)	9 (10.98)	–
**Chemotherapy, n (%)**				
No	700 (96.42)	635 (98.60)	65 (79.27)	*P* < 0.0001
Yes	26 (3.58)	9 (1.40)	17 (20.73)	–
**Surgery, n (%)**				
No	0	0	0	NA
Yes	726 (100.00)	644 (100.00)	82 (100.00)	–

DM (+), patients with distant metastasis; DM (−), patients without distant metastasis.

We compared the characteristics of patients in the metastatic and non-metastatic groups from the SEER database. Thirteen clinicopathological factors were included in our study: age, sex, race, marital status, tumor size, tumor laterality, histological type, tumor grade, T-stage, N-stage, radiotherapy, chemotherapy, and surgery. Patients from the SEER database were categorized into two subgroups: DM (−) (47,899 patients without distant metastases, 92.89%) and DM (+) (3,667 patients with distant metastases, 7.11%). Our analysis revealed that a higher proportion of patients aged ≥ 50 years was observed in the DM (+) subgroup compared to the DM (−) subgroup (*P* < 0.0001); Males exhibited significantly higher metastatic rates than females in DM (+) (*P* < 0.0001); The proportion of White, Asian, or Pacific Islander patients was higher in the DM (+) subgroup compared to the DM (−) subgroup (*P* < 0.0001). Additionally, married patients (2,393/33,974, 7.05%) showed a higher incidence of distant metastasis than single patients (557/8,358, 6.66%; *P* = 0.0131). Regarding renal cancer progression, a greater proportion of patients with tumor sizes larger than 5 cm was observed in the DM (+) group (87.37%) compared to the DM (−) group (34.35%, *P* < 0.0001). The DM (+) subgroup also exhibited significantly higher proportions of certain histological subtypes: 8120/3 (1.91% vs. 0.32%), 8130/3 (0.38% vs. 0.28%), 8310/3 (73.22% vs. 70.50%), and 8312/3 (17.59% vs. 10.81%) compared to DM (−) (*P* < 0.0001); The DM (+) subgroup exhibited a significantly higher prevalence of Grade III–IV disease (histopathological grading), T2–T4 category (tumor extent), and N1–N2 category (regional lymph node involvement) compared to the DM (−) subgroup (*P* < 0.0001); significant disparities in treatment administration (radiotherapy, chemotherapy, surgery) between subgroups (*P* < 0.0001).

### 3.2 Feature variable selection

**FIGURE 2:**
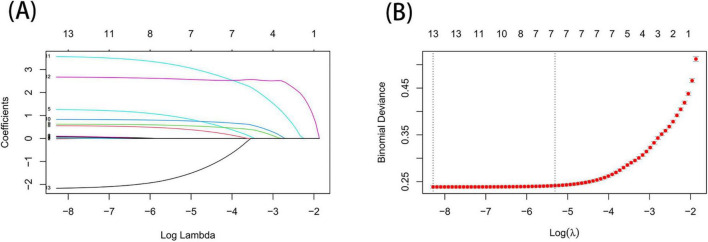
Risk factors for distant metastasis of renal cancer identified by LASSO regression. **(A)** Based on the logarithmic (lambda) sequence, a coefficient profile was created, yielding non-zero coefficients corresponding to the optimal lambda value. **(B)** The process of selecting the optimal value for the parameter λ in the Lasso regression model was performed using cross-validation. The dotted vertical lines indicate the optimal predictors based on the minimum criteria and the 1 standard error of the minimum criteria (1-SE criteria).

As shown in Figure 2, based on Lasso regression analysis, two sets of regularization parameters (λ), λ.min (0.000252) and λ0.1se (0.004947), were determined using 10-fold cross-validation. To optimize the balance between model complexity and generalization, the most parsimonious parameter, λ0.1se, which corresponds to the range within one standard error, was selected as the optimal parameter. Seven significant predictors were identified in the training set: maximum tumor diameter, histological grade, T-stage, N-stage, radiotherapy, chemotherapy, and surgical intervention. Univariate and multivariate logistic regression analyses were conducted on these predictors, with the results summarized in Table 4. Tumor size, grade, T-stage, N-stage, radiotherapy, and chemotherapy were ultimately identified as independent risk factors for distant metastasis in renal cell carcinoma (RCC) patients (*P* < 0.001). Additionally, surgery (OR = 0.14, 95% CI = 0.13–0.16, *P* < 0.001) was found to be an independent protective factor for RCC distant metastasis. Variables with *P* < 0.05 in the multivariate logistic regression analysis were subsequently included in the machine learning model.

**TABLE 4:** Univariate and multivariate logistic regression in patients with distant metastases from renal cancer.

Variables	Univariate logistics			Multivariable logistics		
	OR	95% CI	*P*	OR	95% CI	*P*
Size	13.23	11.98—14.61	*P* < 0.001	3.64	3.16–4.18	*P* < 0.001
Grade	3.59	3.42–3.75	*P* < 0.001	1.76	1.65–1.88	*P* < 0.001
T-stage	3.83	3.67–3.98	*P* < 0.001	1.88	1.76–2.01	*P* < 0.001
N-stage	10.52	9.64–11.48	*P* < 0.001	2.29	2.06–2.54	*P* < 0.001
Radiation	110.73	93.24–131.50,	*P* < 0.001	36.29	29.37–44.83,	*P* < 0.001
Chemotherapy	69.7	63.42–76.59	*P* < 0.001	14.43	12.87–16.17	*P* < 0.001
RX Summ-Surg	0.14	0.13–0.16	*P* < 0.001	0.11	0.09–0.13	*P* < 0.001

Univariate logistic regression analysis revealed that tumor size, tumor grade, T-stage, N-stage, radiotherapy, and chemotherapy were significant risk factors for distant metastasis in renal cancer patients (OR and 95% CI all greater than 1, *P* < 0.05). In contrast, surgical intervention was identified as a protective factor against distant metastasis (OR and 95% CI all less than 1, *P* < 0.05). Multivariate logistic regression analysis confirmed that tumor size, tumor grade, T-stage, N-stage, radiotherapy, and chemotherapy remained independent risk factors for distant metastasis (OR and 95% CI both greater than 1, *P* < 0.05), while surgical intervention, as an independent protective factor, significantly reduced the risk of distant metastasis (OR and 95% CI both less than 1, *P* < 0.05). RX Summ-Surg, surgery.

### 3.3 Model performance evaluation

**FIGURE 3:**
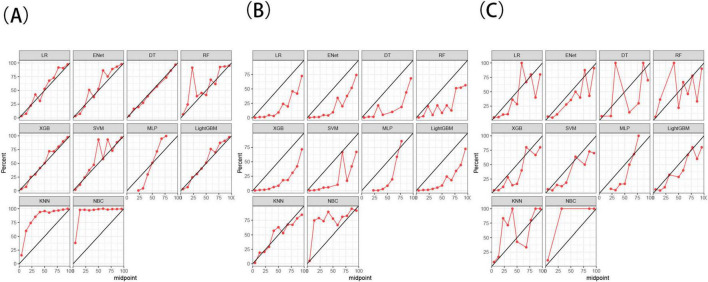
Calibration curves of 10 machine learning methods in the training set **(A)**, test set **(B)**, and external validation set **(C)**. The black diagonal line represents the ideal calibration curve. A calibration curve closer to this line indicates better model calibration.

To build a predictive model for distant metastasis of RCC using ML algorithms, we used seven features (tumor size, tumor grade, T-stage, N-stage, radiotherapy, chemotherapy, and surgery) identified through screening as independent factors. The algorithms used included LR, DT, RF, NBC, KNN, SVM, Enet, MLP, XGB, and LightGBM. To reduce overfitting and select the best model, We conducted 10-fold cross-validation on the training set, evaluating accuracy, precision, recall, F1 score, and AUC for ten ML models (Table 5), and calibration curve plots (Figure 3).

**TABLE 5:** Model performance evaluation metrics for ten machine learning models.

Data set	Model	Accuracy	Precision	Recall	F1 score	AUC
Train	LR	0.887	0.889	0.885	0.887	0.953
	ENet	0.887	0.888	0.886	0.887	0.953
	DT	0.888	0.884	0.893	0.888	0.946
	RF	0.890	0.894	0.885	0.890	0.949
	XGB	0.890	0.897	0.881	0.889	0.957
	SVM	0.887	0.892	0.880	0.886	0.951
	MLP	0.891	0.895	0.886	0.890	0.959
	LightGBM	0.890	0.896	0.881	0.889	0.957
	KNN	0.884	0.935	0.825	0.877	0.938
	NBC	0.884	0.890	0.875	0.883	0.951
Test	LR	0.891	0.387	0.894	0.540	0.950
	ENet	0.891	0.386	0.894	0.539	0.949
	DT	0.885	0.371	0.882	0.522	0.939
	RF	0.896	0.397	0.875	0.546	0.942
	XGB	0.898	0.402	0.874	0.550	0.949
	SVM	0.894	0.393	0.876	0.543	0.948
	MLP	0.896	0.398	0.873	0.547	0.945
	LightGBM	0.898	0.403	0.875	0.552	0.950
	KNN	0.931	0.513	0.821	0.631	0.935
	NBC	0.892	0.388	0.872	0.537	0.944
Validation	LR	0.902	0.612	0.366	0.458	0.749
	ENet	0.898	0.577	0.366	0.448	0.732
	DT	0.888	0.508	0.366	0.426	0.669
	RF	0.902	0.617	0.354	0.450	0.731
	XGB	0.904	0.630	0.354	0.453	0.742
	SVM	0.904	0.625	0.366	0.462	0.706
	MLP	0.897	0.581	0.305	0.400	0.731
	LightGBM	0.905	0.644	0.354	0.457	0.727
	KNN	0.904	0.630	0.354	0.453	0.706
	NBC	0.898	0.587	0.329	0.422	0.719

Test set accuracy is inflated by majority-class dominance. F1 score and AUC better reflect model utility for imbalanced data. The XGB model demonstrates the most balanced and stable performance metrics across all datasets.

**FIGURE 4:**
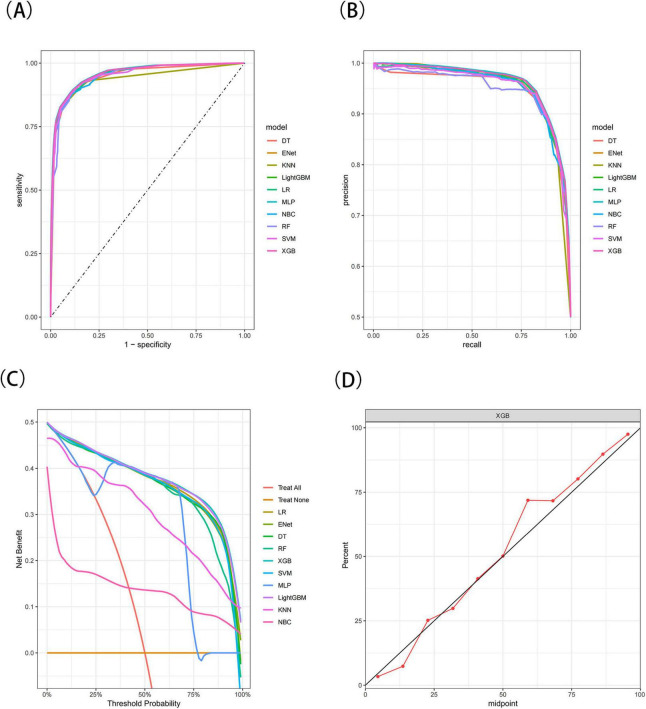
The receiver operating characteristic (ROC) curves **(A)**, Precision-Recall (PR) curves **(B)**, Decision Curve Analysis (DCA) curves **(C)**, and calibration curves **(D)** of the 10 machine learning models in the training set, with calibration curves based on the best model.

**FIGURE 5:**
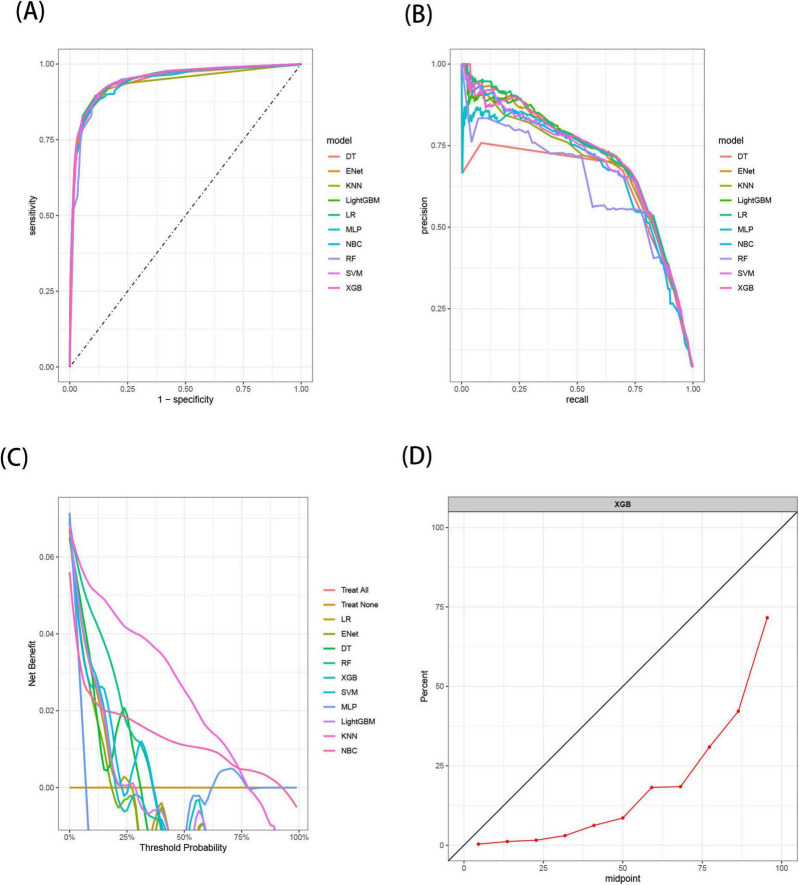
The receiver operating characteristic (ROC) curves **(A)**, Precision-Recall (PR) curves **(B)**, Decision Curve Analysis (DCA) curves **(C)**, and calibration curves **(D)** of the 10 machine learning models in the test set, with calibration curves based on the best model.

**FIGURE 6:**
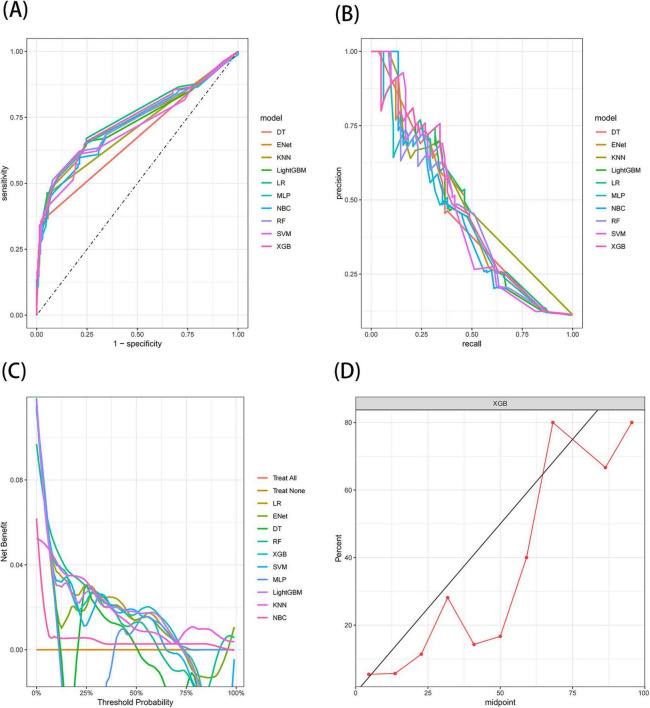
The receiver operating characteristic (ROC) curves **(A)**, Precision-Recall (PR) curves **(B)**, Decision Curve Analysis (DCA) curves **(C)**, and calibration curves **(D)** of the 10 machine learning models in the external validation set, with calibration curves based on the best model.

**FIGURE 7:**
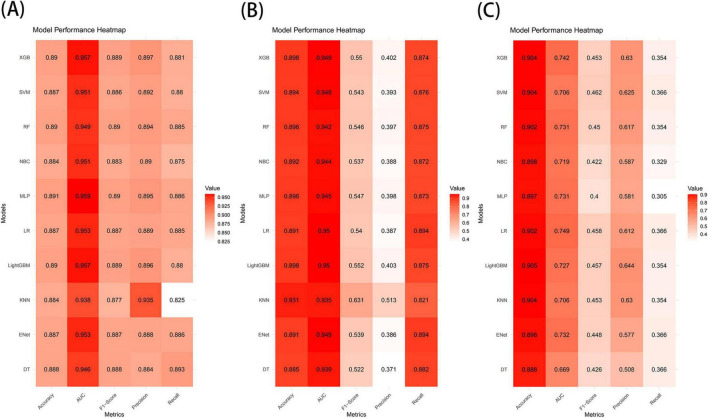
Predictive performance of 10 models in the training set **(A)**, test set **(B)**, and external validation set **(C)**.

The results demonstrated that the XGB model exhibited the most stable performance and superior discriminative ability in the validation set. Figures 4–6 represent the ROC curves, PR curves, DCA curves, and calibration curves for all 10 models across the training, test, and external validation sets. The XGB model consistently delivered strong and stable performance across all datasets, outperforming the other models in terms of discriminative power. Furthermore, the heatmap analysis (Figure 7) offers a comprehensive multidimensional assessment, providing a clearer and more detailed overview of the model’s performance. Following a thorough evaluation of the models across the three datasets, we conclude that the XGB model demonstrates balanced and robust performance in predicting distant metastasis in RCC patients, thus making it the optimal model.

### 3.4 Interpretability of the model

**FIGURE 8:**
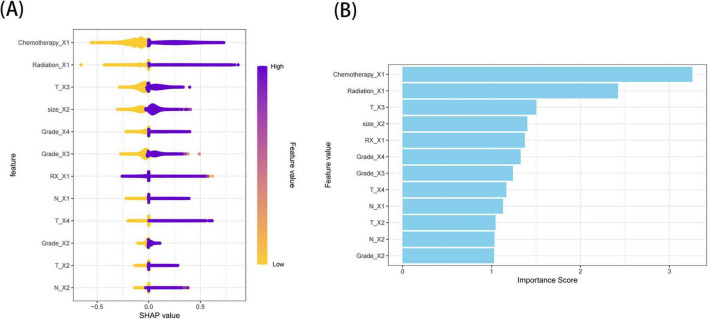
Relative importance of variables based on SHAP for XGB prediction model. Where **(A)** illustrates the SHAP value distribution of features and **(B)** shows the feature importance scores visualized as a bar plot.Chemotherapy_X1 indicates receipt of chemotherapy, Radiation_X1 indicates receipt of radiotherapy, T_X3 represents tumor stage T3, size_X2 indicates a tumor size greater than 5 cm, RX_X1 indicates receipt of surgical treatment, Grade_X4 represents tumor grade IV, Grade_X3 represents tumor grade III, T_X4 represents tumor stage T4, N_X1 represents tumor stage N1, T_X2 represents tumor stage T2, N_X2 represents tumor stage N2, and Grade_X2 represents tumor grade II. SHAP, Shapley’s Additive Explanation; RX, RX Summ-Surg (surgery).

Shapley’s Additive Explanation values were employed to interpret the XGB model. Generally, a higher SHAP value for a feature correlates with an increased probability of the target event occurring. The study results indicated that undergoing chemotherapy was the most significant variable, followed by receiving radiotherapy, having stage T3 disease, possessing a tumor size greater than 5 cm, undergoing surgery, Grade IV, Grade III, stage T4, stage N1, stage T2, stage N2, and Grade II (Figure 8).

### 3.5 Online calculator for predicting distant metastasis in RCC

**FIGURE 9:**
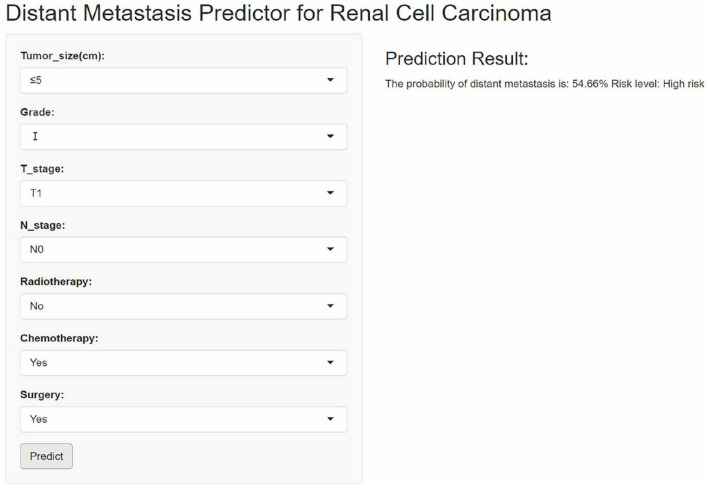
An online web-based calculator for predicting distant metastasis of renal cell carcinoma.

Although the XGB model outperformed other machine learning models, its complexity and limitations in interpretability pose challenges for clinical application. To enhance its clinical utility, we developed an interactive web-based calculator based on the XGB model. This tool allows clinicians to input variables via interactive fields to estimate the probability of distant metastasis in RCC patients. Figure 9 displays a screenshot of the online web calculator. The web calculator can be accessed via the following link: https://houzhao11.shinyapps.io/DM_Predictor/.

## 4 Discussion

The majority of RCC patients are diagnosed with localized disease, while a small proportion present with metastases at onset. However, up to 30% experience distant metastasis following radical resection (27, 28), and the exact molecular mechanisms remain poorly understood. The mRCC patients usually have poor clinical survival, despite the introduction of numerous new targeted and immunotherapeutic agents, patients inevitably develop resistance to these treatments (29, 30). A study by Pereira et al. (31) found that positron emission tomography-computed tomography (PET/CT) offers significantly higher specificity and negative predictive value than CT scans in detecting metastasis and recurrence in RCC patients. However, due to its high cost and the potential risk of radiation exposure, PET/CT is not commonly used for routine screening of distant metastases (32). Consequently, developing a clinical prediction model to identify high-risk RCC patients is crucial.

This study successfully developed and validated an interpretable machine learning model (XGBoost) for predicting the risk of distant metastasis in RCC patients, utilizing data from the SEER database and a single-center cohort in China The results demonstrate that the XGBoost model exhibits balanced and stable predictive performance in both the internal test set and external validation set, outperforming traditional statistical methods, and revealed the non-linear associations of key drivers such as chemotherapy, radiotherapy, and T-stage through the SHAP analysis. This result provides a new tool for early detection and individualized intervention of RCC metastasis, as well as a data-driven perspective for the exploration of tumor biological mechanisms.

The innovation of the present model, compared to previous studies, is evident in three key aspects: first, by integrating data from the SEER cohort (*n* = 51,566) and a Chinese validation cohort (*n* = 726), cross-regional and multi-center validation of the model’s generalization ability was achieved, although external validation performance (AUC = 0.742) declined compared to internal testing (AUC = 0.949), this discrepancy may stem from marked skews in both tumor histologic subtype distribution (93.4% being ICD-O-3 type 8310/3) and treatment patterns (100% surgical intervention) within the validation cohort, suggesting that model requires further optimization for heterogeneous populations. Additionally, the relatively small sample size of the Chinese cohort may exacerbate the impact of class imbalance on the model’s generalizability. Second, the category imbalance problem of distant metastasis samples was effectively alleviated by the SMOTE technique. Third, the web-based calculator developed for the first time transformed the XGBoost model into a visualization tool, providing clinicians with a dynamic risk assessment interface, which is in line with the requirement of “algorithmic transparency” in the International Guidelines for the Application of Artificial Intelligence in Medicine (33).

It is crucial to emphasize that the performance of external validation (AUC = 0.742) was lower than that of internal testing (AUC = 0.949). This inconsistency can primarily be attributed to differences in surgical management structures across study groups and the histological heterogeneity present. Specifically, Surgical Intervention Bias: The Chinese validation cohort exclusively included patients who underwent surgical treatment, resulting in a surgery rate of 100%. In contrast, the SEER dataset encompasses a heterogeneous real-world population that includes non-surgical management of advanced cases. This bias arises from variations in clinical practices and inherently limits the generalizability of the model. Dominance of Histological Subtypes: The validation cohort exhibited a significantly higher proportion of clear cell carcinom, while the SEER dataset comprised a diverse range of histological types. Different subtypes may demonstrate distinct treatment responses and prognostic trajectories, contributing to instability in the model’s performance. T-stage Distribution: The proportion of early-stage tumors is notably higher in the Chinese cohort (87.1% T1) compared to the SEER dataset (68.4%). This discrepancy may reflect a referral pattern that is biased toward localized disease.

External validation is crucial for accurately assessing the reliability of risk prediction models; failure to conduct appropriate external validation can lead to misinterpretation of model performance (34, 35). In this study, we acknowledge the presence of selection bias within the validation cohort, which demonstrates certain differences when compared to the SEER dataset. The lack of adequate external validation may limit the model’s applicability in specific populations. Consequently, to mitigate the selection bias present in the current analysis, we plan to strengthen our external validation efforts in future studies to enhance the model’s reliability. We will consider incorporating a broader patient sample, including data from multiple centers across China and European databases, such as the European RECUR database, to achieve more comprehensive external validation.

Shapley’s Additive Explanation analysis revealed two levels of predictive mechanisms: Treatment-related factors. The analysis revealed that chemotherapy and radiotherapy emerged as the most significant contributors to metastatic risk, a finding that aligns closely with the immunomodulatory dynamics of the RCC microenvironment. Mechanistically, chemotherapy may potentiate immunogenicity by inducing tumor cell release of neoantigenic epitopes (36), while radiotherapy could paradoxically activate pro-metastatic inflammatory cytokine cascades (37), underscoring the dual-edged role of conventional therapies in modulating metastatic propensity; Markers of tumor heterogeneity: Tumor size > 5 cm and high-grade pathological classification (Grade III–IV) are significant factors in the distant metastasis of renal cancer, suggesting that large-volume tumors may induce epithelial-mesenchymal transition (EMT) through mechanical stress, whereas high-grade is associated with vasculogenic mimicry (38, 39). It is noteworthy that surgical intervention was identified as a protective factor; however, its protective efficacy was markedly attenuated in the high-risk metastasis subgroup. This phenomenon may be attributed to the preoperative dissemination of occult micrometastases, necessitating further validation through dynamic monitoring of circulating tumor DNA (ctDNA) (40).

Despite the rigorous study design, this study has the following limitations: Data level: Firstly, the SEER database lacks radiogenomic, genomics, and immunotherapy data, which limits the integration of multimodal information. Additionally, the absence of comprehensive details regarding radiotherapy and chemotherapy protocols restricts our understanding of treatment impacts. Furthermore, the ethnic homogeneity (100% Asian) in the validation cohort may affect the applicability of the model in different populations. To address these gaps, future studies should integrate multi-source datasets (e.g., National Cancer Database, institutional electronic health records) to enrich the SEER dataset with multimodal information and specific treatment regimens. This integration will facilitate a more nuanced assessment of how distinct treatment modalities and regimens influence distant metastasis in renal cell carcinoma, ultimately enhancing the model’s applicability across various population groups. Methodological level: while SMOTE alleviates class imbalance, it may induce synthetic sample bias (41); SHAP interpretation provides only static feature importance and fails to reveal time-dependent metastasis-driven mechanisms (42). Additionally, this study primarily focused on traditional machine learning models. Future research should explore the potential of deep learning architectures like TabNet, particularly on larger datasets. Such models could provide valuable insights into improving predictive accuracy while maintaining interpretability. To address this limitation, future research plans to implement a time-based interpretability framework designed to capture the spatiotemporal effects of treatment characteristics on the mechanisms of metastasis. Our approach will specifically encompass: Dynamic Time Series Modeling: We will integrate Long Short-Term Memory (LSTM) networks with XGBoost to effectively analyze longitudinal data, including sequential tumor markers and treatment regimens. To enhance our analysis, we will collaborate with multi-center medical institutions to obtain comprehensive treatment timelines that include specific start dates for adjuvant therapies and detailed drug regimens. This integration will enable our model to identify and learn time-dependent patterns associated with risk factors, thereby providing deeper insights into how the timing of treatments influences metastatic outcomes. Clinical translational level: the current model relies mainly on traditional clinical metrics (such as tumor size and staging) but does not incorporate some of the most recent detection metrics (such as PD-L1 protein level and VHL mutation status) (43). These metrics can help to determine the patient’s response to precision drug therapies (such as targeted agents and immunotherapies), whereas omitting them may lead to the model’s inability to accurately predict metastatic risk in patients receiving novel therapies. In the future, we can use single-cell sequencing technology to analyze the genetic changes of each cancer cell at different stages and draw a dynamic “genetic map” of tumors from early stage to metastasis; at the same time, we build an intelligent risk warning tool, embedding the model into the hospital’s electronic medical record system, automatically integrating the patient’s latest examination data (such as serum biomarkers, and radiographic findings), enabling real-time metastasis risk stratification, and assisting doctors in adjusting the treatment plan.

This study confirms the practical value of interpretable ML in predicting RCC metastasis. The XGB model developed in this study not only surpasses the limitations of conventional prognostic tools but also provides clinical interpretability through the SHAP framework. The XGB model provides a new tool for individualized prediction of the risk of distant metastasis in renal cancer patients, and by identifying high-risk patients, clinicians can formulate more active follow-up and treatment strategies to improve patients’ prognosis. Future directions include prospective multicenter validation to assess the model’s dynamic predictive ability, further optimization of its performance, and integration into clinical decision support systems to enhance precision oncology strategies for RCC.

## 5 Conclusion

This study confirms the practical value of interpretable machine learning in RCC metastasis prediction, and the XGBoost model constructed by it not only overcomes limitations of conventional prognostic tools but also provides clinical interpretability through the SHAP framework. The XGBoost model provides a new tool for individualized prediction of the risk of distant metastasis in renal cancer patients, and by identifying high-risk patients, clinicians can formulate more active follow-up and treatment strategies to improve patients’ prognosis. Future directions include prospective multicenter validation to verify the dynamic predictive ability of the model, continue to optimize the model performance, and integrate it into the clinical decision support system, to assist in the precision oncology strategies for RCC.

## Data Availability

The raw data supporting the conclusions of this article will be made available by the authors, without undue reservation.

## Footnotes

1
http://seer.cancer.gov/about/
