# Supplementary material for: Explainable machine learning for predicting distant metastases in renal cell carcinoma patients: a population-based retrospective study
Source: Front Med (Lausanne). 2025 Jul 29;12:1624198. doi: 10.3389/fmed.2025.1624198 (PMC12339556; doi:10.3389/fmed.2025.1624198)
Supplement: Supplementary file 1 [file Table_1.docx]

**Best parameters for each machine learning algorithm model**

| **Model** | **Parameters** |
| --- | --- |
| DT | cost_complexity=0.0000893,tree_depth=7,min_n=9 |
| RF | mtry=2,trees=500,min_n=20 |
| NBC | smoothness=0.5,laplace=1 |
| KNN | neighbors=11 |
| SVM | cost=0.0312,rbf_sigma=0.00316 |
| Enet | penalty=0.00599,mixture=0.25 |
| MLP | hidden_units=24,penalty=0.001,epochs=150 |
| XGB | mtry=2,min_n=6,tree_depth=2,learn_rate=0.0885,loss_reduction=0.226,sample_size=0.913 |
| LightGBM | mtry=4,trees=469,min_n=9,tree_depth=2,learn_rate=0.0470,loss_reduction=0.190 |
